# Prevalence, bacterial etiology, and antimicrobial susceptibility patterns of urinary tract infections among pregnant women in rural West Amhara, Ethiopia

**DOI:** 10.1038/s41598-025-25655-4

**Published:** 2025-11-07

**Authors:** Mulatu Melese Derebe, Unmesha Roy Paladhi, Firehiwot Workneh, Abaineh Munshea, Gizachew Yismaw, Kalkidan Yibeltal, Nebiyou Fasil, Alemayehu Worku, Tsehaynesh Gebreyesus, Wudu Tafere, Alem Tsega, Parul Christian, Rose L. Molina, Blair J. Wylie, Yemane Berhane, Anne C C Lee

**Affiliations:** 1 Health Biotechnology Division, Institute of Biotechnology, Bahir Dar University, Bahir Dar, Ethiopia; 2 Amhara Public Health Institute, Bahir Dar, Ethiopia; 3 Global Alliance for Infant and Maternal Health, Department of Pediatrics, Warren Alpert Medical School, Brown University, Providence, RI USA; 4 Department of Epidemiology and Biostatistics, Addis Continental Institute of Public Health, Addis Ababa, Ethiopia; 5 Department of Reproductive Health and Population, Addis Continental Institute of Public Health, Addis Ababa, Ethiopia; 6 Department of Global Health and Health Policy, Addis Continental Institute of Public Health, Addis Ababa, Ethiopia; 7 Department of Human Development, Teachers College, Columbia University, New York, USA; 8 Beth Israel Deaconess Medical Center, Boston, MA USA

**Keywords:** UTI, Uropathogen, Pregnancy, Antimicrobial susceptibility, Urinary tract infection, Epidemiology, Bacteriology

## Abstract

Urinary Tract Infections (UTIs) in pregnant women can lead to pyelonephritis and preterm birth. Our objective was to assess UTI prevalence, etiology, antimicrobial susceptibility, and risk factors among pregnant women receiving antenatal care in rural Amhara, Ethiopia. From a pregnancy cohort in West Gojjam and South Gondor, we consecutively enrolled 604 women from 12 health centers and screened for UTI at ≤ 24 weeks gestational age from August 2020 to June 2022. Women provided urine samples for culture, dipstick, and antibiotic susceptibility testing. Medical history and demographic data were also collected from enrolled participants. We conducted descriptive statistics to describe UTI prevalence and logistic regression to examine risk factors for UTIs. The overall prevalence of UTI was 3.5% (21/604, 95% CI = 2.0%-4.9%), among which 43% were symptomatic and 57% were asymptomatic. Common uropathogens were *Escherichia coli* (57.1%), *Klebsiella pneumoniae* (14.3%), and *Enterococcus faecalis* (14.3%). Among all isolates, resistance was high for ampicillin (66.7%) and amoxicillin-clavulanic acid (40.0%). The majority of isolates (76.2%) were susceptible to nitrofurantoin, cotrimoxazole, and cefpodoxime. Maternal age > 20 years was significantly associated with lower odds of UTI (aOR = 0.29, 95% CI = 0.09–1.00, *p* = 0.05). Urine dipstick (nitrite or leukocyte esterase) had low sensitivity (37.5%) but higher specificity (93.9%) to identify positive culture. In conclusion, in rural communities in Amhara, there is high resistance to first-line antibiotics used to treat UTI in pregnancy and a need for accurate, low-cost UTI diagnostic methods. Increasing access to effective treatment of UTI in low- and middle-income countries is needed to improve maternal and pregnancy outcomes.

**Supplementary Information:**

The online version contains supplementary material available at 10.1038/s41598-025-25655-4.

## Introduction

Urinary tract infections (UTIs) pose a significant public health problem, with more than 150 million global annual incident cases^1^. Pregnancy is a critical period that increases the likelihood of UTIs due to various risk factors, such as hormonal and physiological changes in the urinary tract, and the growing uterus impairing urinary flow. These factors increase the susceptibility of pregnant women to infections, which may lead to a higher risk of morbidity and underscore the importance of effective management and treatment^2^. UTIs during pregnancy can start from asymptomatic bacteriuria (ASB) and acute cystitis and progress to more serious conditions such as pyelonephritis^3^. The UTI prevalence among pregnant women varies across study populations, with a global prevalence reaching as high as 23.9%^4^. A systematic review reported UTI prevalence in Africa ranging from 6.2% to 47.5%^5^. Studies in Ethiopia reported a lower prevalence between 9.8%−26.6%^6,7^, with ASB affecting 2% to 7% of these pregnant women^8^. In pregnant women, UTIs may be associated with maternal complications including pregnancy-induced hypertension, preeclampsia, transient renal insufficiency, sepsis, anemia, and, in rare cases, death^3,9^. Additionally, adverse birth outcomes associated with UTIs in pregnancy include preterm birth, intrauterine fetal growth restriction, and low birth weight^9^.

Pregnant women are exposed to various risk factors that can lead to UTIs^3,10^. Previous studies in high-income countries and LMICs like Ethiopia have identified associated factors for pregnancy-related UTIs. These include a prior history of UTIs, diabetes mellitus, high parity, low socioeconomic status, immunosuppression, gestational age, advanced maternal age, and late presentation for prenatal care^11,12^. This underscores the importance of determining the risk factors for UTIs in these settings to target better screening, prevention, and treatment strategies to mitigate the negative impacts of UTIs during pregnancy^13^. It is essential for healthcare providers to effectively identify, diagnose, and treat UTIs in pregnant women to prevent adverse maternal and birth outcomes.

The common bacterial etiology of UTIs among pregnant women includes *Escherichia coli (E. coli)*,* Klebsiella pneumoniae (K. pneumoniae)*, *Proteus mirabilis (P. mirabilis)*, *Enterobacter species*, *Staphylococcus saprophyticus (S. saprophyticus)*, and group B beta-hemolytic Streptococcus^14^. Among these, *E. coli* is a leading cause of UTIs in Africa and Asia during pregnancy^5^. The emergence of antimicrobial resistance (AMR) in UTI management leads to public health problems worldwide, especially in developing countries with limited diagnostic and healthcare services^15^. Thus, examining antibiotic susceptibility patterns is important to guide appropriate antibiotic treatment in local populations and contexts. However, AMR testing is often not done in low- and middle-income countries (LMICs) settings due to cost and resource limitations. A systematic review reported that most uropathogens in Asia and Africa showed high resistance to ampicillin (67.2%) with relative sensitivity to nitrofurantoin (65%), ciprofloxacin (71.2%), and ceftriaxone (74.1%)^5^. A meta-analysis conducted among pregnant women in Ethiopia showed that *E. coli* was the predominant isolated pathogen and exhibited high resistance to amoxicillin (81%), followed by amoxicillin-clavulanic acid (80%). In addition, the second most significant pathogen was *Klebsiella* species, which had higher resistance to ampicillin (76%), and both pathogens showed lower resistance to Nitrofurantoin and ceftriaxone^16^.

The World Health Organization (WHO) antenatal care guidelines recommend a single screening by urine culture and treatment based on antimicrobial susceptibility testing (AST)^13^. Implementing this strategy in LMICs like Ethiopia remains challenging due to the high costs and logistical complexities associated with performing urine culture. In most rural districts of Ethiopia, diagnosis of UTIs among pregnant women is primarily limited to urine dipstick tests, with microbiological laboratory facilities for urine culture and AST often unavailable^15^. Consequently, empirical treatment is common, resulting in the overuse of antibiotics. This approach contributes to the development of resistance to commonly prescribed medications for treating UTIs in pregnant women^5^. Most existing studies on UTIs among pregnant women in Ethiopia, including those in the study area, have focused on hospital-based data primarily from urban areas, neglecting rural health facilities where many women receive antenatal care (ANC). The lack of published data regarding the prevalence of UTIs and AST among pregnant women in rural districts underscores the need for more inclusive research in Ethiopia. Therefore, this study aimed to assess the prevalence, etiology, and associated risk factors of UTIs and describe the antimicrobial susceptibility patterns of bacterial isolates among pregnant women receiving ANC in rural communities of Amhara, Ethiopia.

## Methods

### Study design and settings

The Enhancing Nutrition and Antenatal Infection Treatment (ENAT) study (ISRCTN Registry: ISRCTN15116516) was conducted from August 2020 to June 2022 in the rural districts of the West Gojjam and South Gondar zones, in Amhara, Ethiopia^17^. The ENAT study was conducted in 12 health centers selected based on accessibility, ANC patient volume, and proximity to the regional reference laboratory for sample transportation and microbiological diagnosis. Six of the study sites were randomized to receive an “Enhanced Nutrition Package” (ENP) intervention and the other six received routine nutrition care. Women in the ENP arm received iodized salt and iron-folic acid supplements, and women with a middle-upper arm circumference (MUAC) < 23 cm received a micronutrient-fortified Balanced Energy Protein (BEP). In all 12 sites, individual women were randomized to receive either the “Enhanced Infection Management Package” (EIMP) intervention or standard infection care. Women in the EIMP arm received screening and treatment for UTI/ASB and sexually transmitted infections/reproductive tract infections at enrollment, as well as presumptive deworming in the second trimester, followed by stool screening and treatment at least four weeks later. Those in the standard care arm received syndromic management of diseases and health systems strengthening. The full ENAT study protocol has been publihsed^17^.

In the current sub-study and secondary analysis, we use prospective data from the EIMP study arm of the parent ENAT study during the period of UTI screening to examine the prevalence and associated risk factors of UTI among pregnant women within the EIMP arm. UTI screening was stopped in February 2021 due to the unavailability of supplies due to COVID-19 and civil unrest.

### Study population

In this sub-cohort, we include pregnant women enrolled in the ENAT study with a viable pregnancy identified at ≤ 24 weeks gestation who were consecutively enrolled into the EIMP study arm during the period of UTI screening (August 2020- February 2021).

### Study procedures and data collection

A study nurse explained the procedures to potential participants presenting for their ANC visit and obtained consent for the parent study. Study nurses collected data through verbally administered structured questionnaires, including sociodemographic indicators, medical and obstetric history, morbidity symptoms, and nutritional intake. Nurses were trained and standardized to measure maternal anthropometrics using standard operating procedures (Intergrowth 21)^18^. Data was entered into a tablet using the Survey Solutions platform (World Bank, https://mysurvey.solutions/en/*).*

### Sample collection and processing

The study nurses and health center laboratory technicians provided participants with detailed instructions on how to collect a clean-mid-catch midstream sterile urine sample. Job aids posted on the wall of the laboratory and available at the tables were used to orient the mother on how to collect and transport the sample to the lab without contamination. A sterile container was used to collect a 20–30 ml clean catch midstream urine sample. A portion of the urine sample was poured into another container and used for the dipstick test. The rest of the sample was transferred to a boric acid-containing vacutainer for transport and storage at room temperature until processing (Beckton Dickinson Urine collection kit: BD 364956). The BD sample collection tube allowed for preserving the urine for up to 48 h at room temperature before processing at Amhara Public Health Institute (APHI). Specimens were transported to the microbiology reference laboratory of APHI for inoculation, culture, and incubation for 48 h. Full biospecimen collection and processing details are described elsewhere^19^. A randomly chosen subset of participant urine samples was also tested using a urine dipstick. After immersion of the dipstick in urine, the samples were visually assessed by the health center laboratory technician after two minutes against a standard color chart for levels of leukocyte esterase, protein, glucose, nitrite, and ketones. The Combur10-Test M-strip was utilized following the manufacturer’s instructions (Roche) at study health centers.

### Urine culture and antimicrobial susceptibility test

At APHI, the urine specimens were inoculated onto Blood agar and MacConkey plates by using a calibrated 0.001 ml (1 µl) inoculating loop and then incubated aerobically at 37 °C for 48 h. The growth of bacterial pathogens was inspected and graded for the presence of significant bacteriuria. The quantification of the colony was done by multiplying the colony count by 1000 to estimate the number of bacteria per ml of urine (CFU/ml). Urine culture demonstrating significant bacteriuria was further identified by its colony characteristics. The identification and Antimicrobial Sensitivity Test (AST) were conducted by using the Vitek-2 compact method with the Biomet Rieux supply kits GN-71 and GP-71 for identification and AST-GN71 and AST-GP 71 for AST (RIF413 402) for both Gram-negative and Gram-positive bacteria, respectively. Well-isolated bacterial colonies were emulsified with 3 ml of 0.45% saline. The inoculum turbidity was adjusted to match 0.5 McFarland standard solutions. For AST of Gram-negative and Gram-positive bacteria, 145 µl and 280 µl volumes of suspension, respectively, were taken from the original suspension. Identification and antimicrobial susceptibility testing cards were loaded into the VITEK-2 Compact software to determine the bacterial species and their AST profiles. The drugs used in the susceptibility test were amoxicillin-clavulanic acid (AMC), ampicillin (AMP), cefazolin (CFZ), cotrimoxazole (COT), cefpodoxime (CPD), and nitrofurantoin (NFT).

### Definitions of outcome

The outcome assessed in this study was urinary tract infection (UTI). High-burden bacterial growth was defined as bacteriuria of ≥ 10^5^ CFU/mL of urine of a single uropathogen and intermediate growth as bacteriuria with ≥ 10^3^ to < 10^5^ CFU/mL of a single uropathogen. Contaminated samples were defined as bacterial growth of > 2 microorganisms or growth of a non-urinary tract pathogen based on the ASM standard^20^. We defined symptomatic UTI as women with ≥ 10^3^ bacteriuria with UTI signs and symptoms, including dysuria, urinary frequency, hematuria, fever, abdominal pain, flank pain, and the urgency of urination. Asymptomatic Bacteriuria (ASB) was defined as bacterial colony counts ≥ 10^5^ CFU/mL from urine samples collected from a person without UTI signs and symptoms. Any UTI was defined as both a symptomatic UTI and ASB.

### Quality assurance

The study nurses and laboratory technicians at each of the study sites were trained in urine sample collection, short-term storage, transportation, and long-term storage. MMD provided intensive supervision and observed sample collection and transportation. The SOPs of the microbiology laboratory at APHI were followed. The sterility of the media was checked by overnight incubation at 37 °C before inoculation, and Quality Control (QC) strain bacteria, such as *Staphylococcus aureus* (*S. aureus*) (ATCC strain 25923), *P. aeruginosa* (ATCC 27853), and *E. coli* (ATCC strain 25922) were used to check the growth support of the media. The VITEK-2 software used for QC testing automatically read the expiration date and card type information. All the data were collected on tablet computers into Survey Solutions and checked for completeness.

### Statistical analysis

We conducted basic descriptive statistics of UTI prevalence, uropathogen distribution, and antimicrobial susceptibility patterns. We used bivariable and multivariable logistic regression modeling to assess factors associated with UTI. The potential risk factors based upon literature review and included: age, education, partner education, occupation, household size, cow/ox ownership, land ownership, drinking water source, toilet type used, parity, gestational age, MUAC, and BMI. All potential associated factors were assessed using bivariable logistic regression, and those variables with *p* < 0.25 were eligible for inclusion in the multivariable model^21^. Due to the high collinear relationship between parity and age, only age was retained in the adjusted model.

The diagnostic accuracy of the urine dipstick test (determined by nitrite and leukocyte esterase) was compared to that of urine culture, which is considered the gold standard. The accuracy of the test was assessed by calculating its sensitivity, specificity, positive predictive value (PPV), negative predictive value (NPV), and positive (+ LR) and negative (− LR) likelihood ratios. The analysis focused on nitrite positivity (NIT+), leukocyte esterase positivity (LE+), either test being positive (NIT + or LE+), and the combination of both tests being positive (NIT + and LE+) related to positive urine results. All analyses were conducted via STATA version 18.0 (StataCorp LP, College Station, TX).

### Ethics approval

The ENAT study protocol was evaluated and approved by the Institutional Review Boards of Mass General Brigham (2018P002479), USA in March 2019, and the Addis Continental Institute of Public Health (001-A1-2019), Addis Ababa, Ethiopia, in February 2019. We received approval and an official support letter from the Amhara Regional State Public Health Institute. Additionally, permissions were obtained from the woredas and the 12 study health centers. Written informed consent was obtained from the study participants (pregnant women) before initiation of study procedures or data collection. All methods were performed in accordance with the relevant guidelines and regulations.

### Consent to participate

Furthermore, written informed consent was obtained from each research participant before data collection.

## Results

**Fig. 1:**
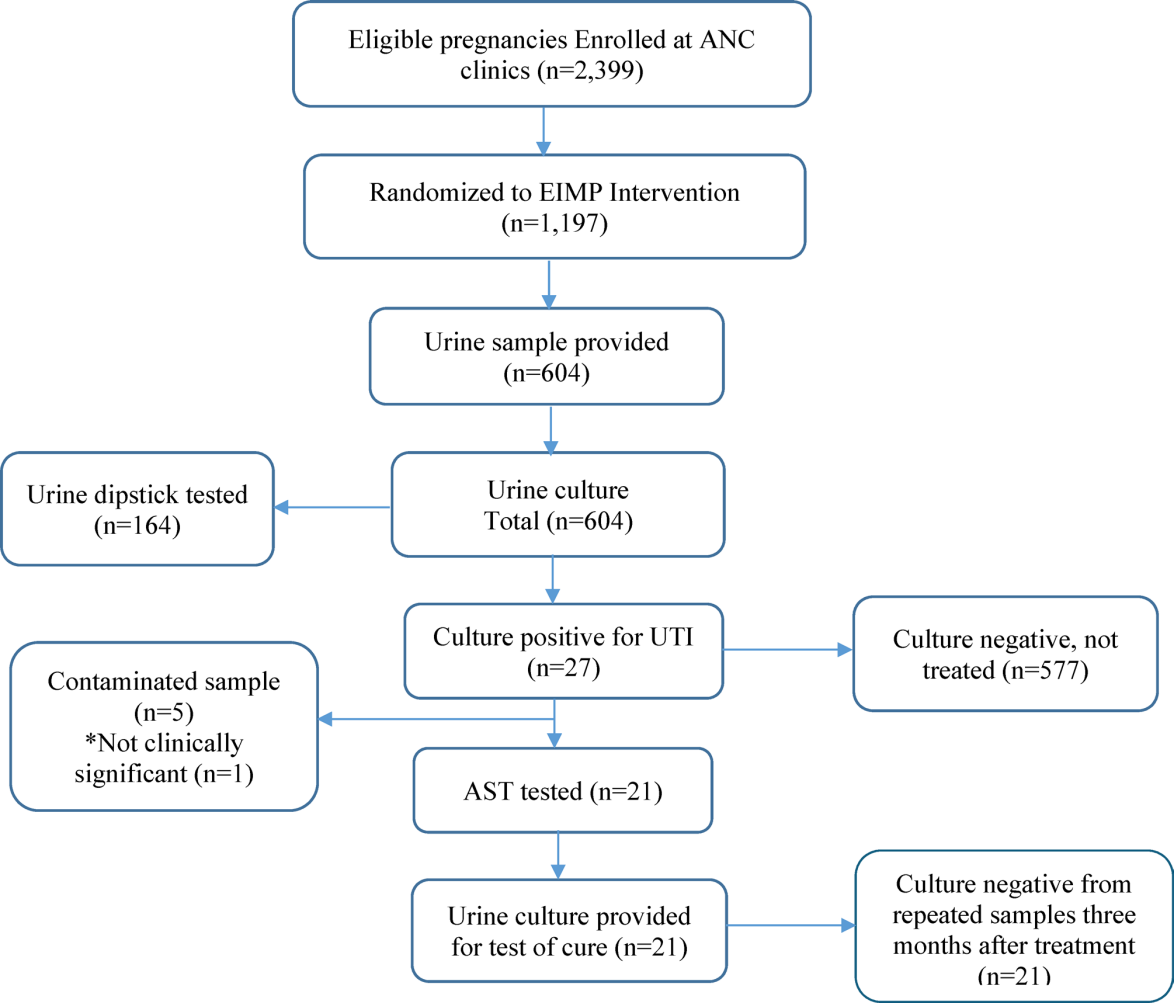
Flow Chart to patient enrollment and specimens’ collection as part of ENAT study intervention among pregnant women receiving ANC visits in rural Amhara, Ethiopia, (2020–2022). *ANC*: Antenatal care; *EIMP*: Enhanced infection management package; *UTI*: Urinary tract infection; *AST*: Antimicrobial sensitivity test. *Not clinically significant, significant growth of UTI bacteria, but asymptomatic; did not fulfill criteria for UTI treatment. Contaminated samples, samples of bacterial growth of > 2 microorganisms, or growth of non-urinary tract pathogens.

Among the 2,399 women enrolled 1,197 were individually randomized to the EIMP intervention (Fig. 1). A total of 604 women provided samples and were screened for urine by culture during the urine screening period. Among the 604 samples, 27/604 (4.5%) showed bacterial growth, 577/604 (95.5%) had no growth, and five were contaminated. Of the 27 culture-positive samples, 21 were identified as significant uropathogens 21/604 (3.5%) and further tested for AST (Fig. 1).

### Prevalence of UTIs

Among the participants included in this study, 22 out of 604 cultures (3.6%; 95% CI:2.2%−5.2%) showed significant or high-burden bacterial growth (Table 1). Asymptomatic bacteriuria (ASB) accounted for 13/22 (59.1%) of cases, and 9/22 (40.9%) were symptomatic (Table 1). The 21 women were treated with selected antibiotics based on antimicrobial susceptibility patterns, and follow-up testing confirmed that all treated patients were cured. Of the 22 participants exhibiting growth, one case did not meet criteria for treatment, given that the mother was asymptomatic and had low burden of growth (Fig. 1; Table 1).

**Table 1:** Prevalence of utis among pregnant women receiving ANC visits in the rural West Amhara, Ethiopia, (2020–2022).

Burden of bacterial growth	Total (*N* = 604) *n* (%)	Symptomatic UTI* (*N* = 9) *n*	Asymptomatic UTI* (*N* = 13) *n*
No growth	577 (95.5)	-	-
High burden growth (> = 100,000 CFU/ml)	20 (3.3)	8.0	12.0
Significant growth (10,000–99,999 CFU/ml)	2 (0.3)	1.0	1.0
Contaminated sample**	5 (0.8)	-	-
Total	604	9.0	13.0

*Symptomatic UTIs were defined as women with chief complaints of dysuria, urinary frequency, hematuria, abdominal pain, fever, or flank pain. *Asymptomatic UTIs were defined as women with significant or high-burden bacterial growth without UTI symptoms. **Samples were considered contaminated when there was bacterial growth of > 2 microorganisms or growth of non-urinary tract pathogens.

### Sociodemographic characteristics and UTIs

In this study, the median age was 26 years (interquartile range [IQR]: 22–30). Most women were married (583, 97.0%) and self-identified as Orthodox Christians (579, 96.3%). Nearly half had no formal education (278, 46.2%) and worked in agriculture, farming, or as daily laborers (285, 47.4%). Lastly, almost three-quarters of the women were multiparous (429, 71.3%) (Table 2).

**Table 2:** Characteristics of pregnant women attending ANC visits in the rural West Amhara, Ethiopia, (2020–2022).

Variables	Overall (*N* = 604)	No UTI (*N* = 583)	UTI^§^ (*N* = 21)
	n (%)	n (%)	n (%)
Age at enrollment			
< 20	63 (10.4)	57 (9.8)	6 (28.6)
≥ 20	541 (89.6)	526 (90.2)	15 (71.4)
Marital status			
Married	583 (97.0)	564 (97.2)	19 (90.5)
Divorced/separated or never married	18 (3.0)	16 (2.8)	2 (9.5)
Religion			
Orthodox Christian	579 (96.3)	558 (96.2)	21 (100.0)
Muslim	22 (3.7)	22 (3.8)	0 (0.0)
Education			
No education	278 (46.3)	271 (46.7)	7 (33.3)
Primary	181 (30.1)	175 (30.2)	6 (28.6)
Secondary and above	142 (23.6)	134 (23.1)	8 (38.1)
Partner Education			
No education	266 (44.3)	259 (44.7)	7 (33.3)
Primary	187 (31.2)	178 (30.7)	9 (42.9)
Secondary and above	147 (24.5)	142 (24.5)	5 (23.8)
Occupation			
Housewife	192 (32.0)	184 (31.7)	8 (38.1)
Wage occupation	124 (20.6)	121 (20.9)	3 (14.3)
Agriculture/farmer/daily laborer	285 (47.4)	275 (47.4)	10 (47.6)
Household size			
1–2 people	206 (34.1)	196 (33.6)	10 (47.6)
≥3 people	398 (65.9)	387 (66.4)	11 (52.4)
Cow/ox ownership			
No	225 (37.4)	215 (37.1)	10 (47.6)
Yes	376 (62.6)	365 (62.9)	11 (52.4)
Land ownership			
No	187 (31.1)	182 (31.4)	5 (23.8)
Yes	414 (68.9)	398 (68.6)	16 (76.2)
Drinking water source			
Public tap	374 (62.2)	364 (62.8)	10 (47.6)
Other water sources (spring, surface & other)	227 (37.8)	216 (37.2)	11 (52.4)
Toilet type used			
No toilet	202 (33.6)	197 (34.0)	5 (23.8)
Any latrine/toilet	399 (66.4)	383 (66.0)	16 (76.2)
Parity*			
Primiparous	173 (28.7)	163 (28.1)	10 (47.6)
Multiparous	429 (71.3)	418 (71.9)	11 (52.4)
Gestational age at time of urine collection**			
1–12 weeks	118 (19.5)	112 (19.2)	6 (28.6)
13–24 weeks	486 (80.5)	471 (80.8)	15 (71.4)
Undernutrition (MUAC < 23)			
Yes (< 23 cm)	187 (31.8)	178 (31.3)	9 (42.9)
No (> 23 cm)	402 (68.2)	390 (68.7)	12 (57.1)
Undernutrition (BMI)			
Underweight (18.5)	90 (14.9)	84 (14.4)	6 (28.6)
Normal weight (18.5–24.9)	480 (79.5)	465 (79.8)	15 (71.4)
Overweight/obese (> = 25)	34 (5.6)	34 (5.8)	0 (0.0)

§UTI was defined as an infection that affects a part of the urinary system, which includes the kidneys, bladder, ureters, and urethra. *Parity is the number of pregnancies carried out by a female for at least 20 weeks. **Gestational age is the age of a pregnancy taken from the beginning of the woman’s last menstrual period. Missingness: 3, marital status; 3, religion; 3, education; 4, partner education; 3, occupation; 3, household size; 3, cow ownership; 3, land ownership; 3, drinking water source; 3, toilet type used; 2, parity; 15, MUAC < 23; 6, BMI.

### Factors associated with UTIs

Higher Maternal age (> 20yo) was associated with lower odds of UTI in the bivariable analysis, (**OR =** 0.27, 95% CI = 0.10–0.72; although this was of borderline significance in the multivariable model (aOR = 0.29, 95% CI = 0.09–1.00.09.00, *p* = 0.050) (Table 3). No other risk factors were significantly associated with UTI in the bivariable or multivariable analysis.

**Table 3:** Factors associated with UTI among pregnant women attending ANC visits in rural West Amhara, Ethiopia, (2020–2022).

Variables	Bivariable model		Multivariable Model	
	OR (95% CI)	p-value	aOR (95% CI)	p-value
Age at enrollment				
< 20	Ref	-	Ref	-
≥ 20	0.27 (0.10–0.72)	0.009	0.29 (0.09–1.00)	0.05
Education				
No education	Ref.	-	-	-
Primary	1.33 (0.44–4.02)	0.616	1.10 (0.32–3.73)	0.879
Secondary and above	2.31 (0.82–6.51)	0.113	3.72 (0.87–15.77)	0.075
Partner Education				
No education	Ref.	-	-	-
Primary	1.87 (0.68–5.11)	0.222	1.34 (0.42−4.22)	0.617
Secondary and above	1.30 (0.40–4.18)	0.656	-	-
Occupation				
Housewife	Ref.	-	-	-
Wage occupation	0.57 (0.15–2.19)	0.414	-	-
Agriculture/farmer/daily labor	0.84 (0.32–2.16)	0.712	-	-
Household size				
1–2 people	Ref.	-	-	-
≥3 people	0.55 (0.23–1.33)	0.189	1.81 (0.43–7.67)	0.42
Cow/ox ownership				
Yes	0.65 (0.27–1.55)	0.33	-	-
No	Ref.	-	-	-
Land ownership				
Yes	1.46 (0.53–4.06)	0.464	-	-
No	Ref.	-	-	-
Drinking water source				
Public tap	Ref.	-	-	-
Other water sources (spring, surface, and others)	0.54 (0.23–1.29)	0.166	0.44 (0.16–1.20)	0.108
Toilet type used				
No toilet	Ref.	-	-	-
Any latrine/toilet	0.61 (0.22–1.68)	0.338	-	-
Parity				
Primiparous	Ref.	-	-	-
Multiparous	0.43 (0.18–1.03)	0.058	0.47 (0.11–1.90)	0.289
Gestational age at time of urine collection				
1–12 weeks	Ref.	-	-	-
13–24 weeks	0.59 (0.23–1.57)	0.293		
Undernutrition (MUAC < 23)				
No (> 23 cm)	Ref.	-	-	-
Yes (≤ 23 cm)	1.64 (0.68–3.97)	0.27		
Undernutrition (BMI)				
Normal weight (18.5–24.9)	Ref.	-	-	-
Underweight (18.5)	2.21 (0.84–5.87)	0.11	1.85 (0.65–5.26)	0.245

*Bivariable models include age, education, partner education, household size, drinking water source, parity, and undernutrition at enrollment (BMI < 18.5). Abbreviations: OR, odds ratio; aOR, adjusted odds ratio; CI, confidence interval; MUAC, middle- and upper-arm circumference; BMI, body mass index; Ref, reference.

### Etiology of UTI

**Fig. 2:**
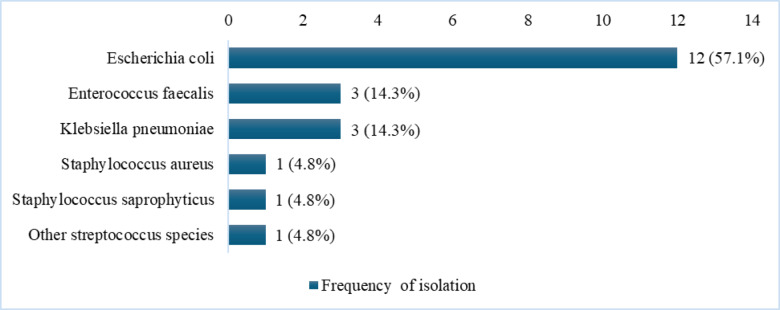
Etiology of uropathogens isolated from pregnant women diagnosed with UTI attending ANC visits in rural West Amhara, Ethiopia, (2020–2022).

Figure 2 presents the bacterial ethiologyof UTIs identified from urine culture among the 21 women who showed either high-burden growth or significant growth and were symptomatic. Six bacterial species were identified. *E. coli* was the most prevalent, accounting for 57.1% (12/21) of the total isolated uropathogens. *K. pneumoniae* (14.3%, 3/21), *Enterococcus faecalis (E. faecalis* (14.3%, 3/21), *S. aureus* (4.8%, 1/21), *S. saprophyticus* (4.8%, 1/21), and other *Streptococcus species* (4.8%, 1/21) were the remaining cases. The majority (58.3%, 7/12) of *E. coli* cases exhibited UTI symptoms. The remaining isolates presented more commonly as asymptomatic bacteriuria: *K. pneumoniae* (66.7%, 2/3), *E. faecalis* (100%, 3/3), *S. saprophyticus* (100%, 1/1), and other *Streptococcus species* (100%, 1/1) (Supplementary Table S1).

### Antimicrobial susceptibility patterns

Table 4 and Supplementary Fig.S1 show the percentages of bacterial isolates that were classified as susceptible, intermediate, or resistant to various antibiotics. Among all of the identified uropathogens, overall rates of antibiotic resistance were high for ampicillin (66.7%, 14/21) and amoxicillin-clavulanic acid (40.0%, 6/15) for Gram-negative uropathogens. The highest overall rates of antimicrobial susceptibility were for cotrimoxazole, cefpodoxime, and nitrofurantoin (each 76.2%, 16/21); followed by cefazolin (61.9%, 13/21); and much lower for ampicillin (28.6%, 6/21). Conversely, by species, *E. coli* had the highest resistance to ampicillin (66.7%, 8/12) and amoxicillin-clavulanic acid (50.0%, 6/12), *E. faecalis* exhibited the highest resistance to ampicillin (66.7%, 2/3) but was fully susceptible to cefpodoxime and nitrofurantoin (100%, 3/3). Furthermore, all the *K. pneumoniae* isolates were resistant to ampicillin (100%, 3/3) but were fully susceptible to cefpodoxime (100%, 3/3).

**Table 4:** Antimicrobial susceptibility patterns of uropathogens among pregnant women attending ANC visits in rural Amhara, Ethiopia (2020–2022).

Bacterial isolate	Total	S/I/R*	Antibiotics					
			Amoxicillin-Clavulanic acid **n (%)**	Ampicillin **n (%)**	Cefazolin **n(%)**	Cotrimoxazole **n(%)**	Cefpodoxime **n(%)**	Nitrofurantoin **n(%)**
*Escherichia coli*	12	S	5 (41.7)	3 (25.0)	6 (50.0)	9 (75.0)	7 (58.3)	9 (75.0)
		I	1 (8.3)	1 (8.3)	2 (16.7)	0 (0.0)	0 (0.0)	3 (25.0)
		R	6 (50.0)	8 (66.7)	4 (33.3)	3 (25.0)	5 (41.7)	0 (0.0)
*Enterococcus faecalis*	3	S	0 (0.0)	1 (33.3)	2 (66.7)	2 (66.7)	3 (100)	3 (100)
		I	0 (0.0)	0 (0.0)	0 (0.0)	0 (0.0)	0 (0.0)	0 (0.0)
		R	0 (0.0)	2 (66.7)	1 (33.3)	1 (33.3)	0 (0.0)	0 (0.0)
*Klebsiella pneumoniae*	3	S	3 (100)	0 (0.0)	2 (66.7)	2 (66.3)	3 (100)	2 (66.7)
		I	0 (0.0)	0 (0.0)	0 (0.0)	0 (0.0)	0 (0.0)	0 (0.0)
		R	0 (0.0)	3 (100)	1 (33.3)	1 (33.7)	0 (0.0)	1 (33.3)
*Staphylococcus aureus*	1	S	0 (0.0)	0 (0.0)	1 (100)	1 (100)	1 (100)	1 (100)
		I	0 (0.0)	0 (0.0)	0 (0.0)	0 (0.0)	0 (0.0)	0 (0.0)
		R	0 (0.0)	1 (100)	0 (0.0)	0 (0.0)	0 (0.0)	0 (0.0)
*Staphylococcus saprophyticus*	1	S	0 (0.0)	1 (100)	1 (100)	1 (100)	1 (100)	1 (100)
		I	0 (0.0)	0 (0.0)	0 (0.0)	0 (0.0)	0 (0.0)	0 (0.0)
		R	0 (0.0)	0 (0.0)	0 (0.0)	0 (0.0)	0 (0.0)	0 (0.0)
*Other streptococcus species*	1	S	0 (0.0)	1 (100)	1 (100)	1 (100)	1 (100)	0 (0.0)
		I	0 (0.0)	0 (0.0)	0 (0.0)	0 (0.0)	0 (0.0)	0 (0.0)
		R	0 (0.0)	0 (0.0)	0 (0.0)	0 (0.0)	0 (0.0)	1 (100)
*Total*	21	S	8 (53.3)	6 (28.6)	13 (61.9)	16 (76.2)	16 (76.2)	16 (76.2)
		I	1 (6.7)	1 (4.8)	2 (9.5)	0 (0.0)	0 (0.0)	3 (14.3)
		R	6 (40.0)	14 (66.7)	6 (28.6)	5 (23.8)	5 (23.8)	2 (9.5)

*S/I/R; S, sensitive; I, intermediate; R, resistant; *ATC*,* Anatomical Therapeutic Chemical*.

Pharmaceutical forms and ATC classification: Amoxicillin-Clavulanic Acid (Oral, J01CR02), Ampicillin (Oral, J01CA01), Cefazolin (Injectable, J01DB04), Cotrimoxazole (Oral, J01EE01), Cefpodoxime (Oral, J01DD13). Nitrofurantoin (Oral, J01XE01).

### Urine dipstick results with diagnostic accuracy compared to culture results

The accuracy of the dipstick test for nitrite and leukocyte esterase was assessed by determining the sensitivity and specificity to identify positive urine culture, which is the gold standard. Nitrite testing (NIT+) alone exhibited a relatively low sensitivity of 28.6% (95% CI = 3.7–70.9) but had a high specificity of 98.6% (95% CI = 95.2–99.8). The positive predictive value (PPV) for nitrite was 50.0% (95% CI = 14.1–85.9), and the negative predictive value (NPV) was 96.7% (95% CI = 94.8–97.9). Leukocyte esterase (LE+) testing alone had a lower sensitivity of 14.3% (95% CI = 0.4–57.9) and a high specificity of 96.2% (95% CI = 91.9–98.6), with a PPV of 14.3% (95% CI = 2.3–54.6). When nitrite-positive or leukocyte esterase-positive results were combined, the sensitivity and specificity were 37.5% (95% CI = 8.5–75.5) and 93.9% (95% CI = 88.2–97.3), respectively. Additionally, compared with culture results, UTI symptoms had a sensitivity of 42.9% (95% CI = 1.7–14.8) and specificity of 64.2% (95% CI = 91.8–99.1) (Table 5).

**Table 5:** Diagnostic accuracy of urine dipstick (nitrite and leukocyte esterase positive) to identify UTI with positive urine culture among pregnant women attending ANC visits in rural Amhara, Ethiopia (2020–2022).

Culture	Total tested *N*	Sensitivity % (95% CI)	Specificity % (95% CI)	PPV % (95% CI)	NPV % (95% CI)	+LR (95% CI)	−LR (95% CI)
NIT+	155	28.6 (3.7–70.9)	98.6 (95.2–99.8)	50.0 (14.1–85.9)	96.7 (94.8–97.9)	21.1 (3.5–128.8)	0.7 (0.4–1.2)
LE+	164	14.3 (0.4–57.9)	96.2 (91.9–98.6)	14.3 (2.3–54.6)	96.2 (94.9–97.1)	3.7 (0.5–27.0)	0.9 (0.7–1.2)
NIT + or LE+	138	37.5 (8.5–75.5)	93.9 (88.2–97.3)	27.3 (10.9–53.4)	96.1 (93.4–97.7)	6.1 (2.0–18.7.0.7)	0.7 (0.4–1.1)
UTI symptoms (dysuria, incontinence, or urgency)	604	42.9 (21.8–66.0)	64.2 (60.1–68.1)	4.1 (2.5–6.7)	96.9 (95.5–97.8)	1.2 (0.7–2.0)	0.9 (0.6–1.3)
UTI symptoms (Syndromic)/NIT/LE	187	50.0 (15.7–84.3)	65.4 (57.9–72.3)	6.1 (3.0–11.7)	96.7 (93.5–98.3)	1.4 (0.7–3.0)	0.8 (0.4–1.5)

LE = leukocyte esterase; NIT = nitrite; UTI = urinary tract infection; PPV = positive predictive value; NPV = negative predictive value; −LR = negative likelihood ratio; +LR = positive likelihood ratio.

## Discussion

The prevalence of UTI, including both symptomatic and asymptomatic bacteriuria, was low (3.5%) among pregnant women in rural Amhara. *E. coli* was the most common uropathogen, followed by Klebsiella and Enterococcus. The majority of isolates were susceptible to nitrofurantoin, cotrimoxazole, and cefpodoxime, while resistance was high for ampicillin. This has relevant implications given that amoxicillin is the current first-line treatment for UTI in pregnant women in Ethiopia. Furthermore, the majority of UTIs were asymptomatic; however, the commonly used and available urine dipstick had very low sensitivity and was an inadequate screening tool for detecting UTIs.

Higher prevalence rates of UTI (9%–30%) have been observed in various LMICs across Asia and Africa^22–26^. In a systematic review of studies in Ethiopia, the prevalence of UTIs among pregnant women ranged from 9.8% to 26.6%^22^. Our study, conducted in Amhara, Ethiopia, was set in a rural, low-risk, and conservative population in outpatient ANC, and reported a relatively lower prevalence. This low prevalence is comparable with studies from Pakistan (4.3%)^27^, Sri Lanka (3.6%)^28^, and Bangladesh (4.0%)^29^. There are several reasons for the differences in local epidemiology, including population characteristics, practices, risk factors, as well as laboratory methods. Other settings may have recruited more urban or high-risk populations, with different patient demographics, socioeconomic factors, hygiene practices, and access to ANC services. Urine collection and storage may also potentially impact urine culture^30^. We did extensive training of laboratory staff and patients to collect urine specimens using aseptic techniques, and rates of urine contamination and growth were lower than in other studies. Furthermore, a recent systematic review and meta-analysis in Ethiopia also identified the Amhara region as having the lowest UTI burden (9.8%)^22^, supporting its lower rates in the region. In this study, the most common pathogen isolated was *E. coli* (57.1%), consistent with studies from Asia and Ethiopia^31–35^, which have also implicated *E. Coli* as a primary uropathogen associated with UTIs. *K. pneumoniae and E. faecalis* (14.3%) were the second most common isolated pathogens, aligning with findings from Nepal and Nigeria^32,36^.

Common risk factors associated with UTI identified in a previous systematic review and other studies include: socio-demographic characteristics (age, education, marital status, occupation, family income) and clinical factors such as history of UTI, parity, gravidity, gestational age, and presence of other infections linked with UTIs^22,37^. In our study population, higher maternal age had a borderline association with lower odds of UTI. This finding may have been attenuated by a single screening point (< 24 weeks of pregnancy) and a small number of UTI cases, but it is consistent with studies in Ghana and Finland, which showed that pregnant women of higher age are less likely to get UTIs compared with younger age groups^38,39^. Some additional borderline associations (maternal undernutrition, parity, education) may have been observed due to similar reasons. UTI screening was conducted only at one time point (at enrollment < 24 weeks of gestation) due to the parent study design. Consequently, we may have systematically excluded women who could exhibit differential UTI burden and risk later in pregnancy, potentially leading to an underestimation of the true burden of UTI and attenuating the observed associations. Other sociodemographic factors that we examined were not significantly associated with UTI, aligning with studies from Tanzania and Sudan^24,40^, and other regions of Ethiopia^37,41–43^. This suggests that targeting urine culture screening based on risk factors may not be the optimal approach to identifying UTI cases in pregnant women in similar settings and populations.

Antimicrobial resistance among uropathogens is a critical challenge for managing UTIs, particularly in LMICs, where there is more limited antibiotic availability for UTIs. This study showed high resistance rates among all pathogens to commonly prescribed antibiotics for UTIs in pregnancy in Ethiopia, such as ampicillin and amoxicillin (66.7%), which is the first-line treatment for dysuria in primary care settings. Similarly, nearly half of the isolates were resistant to amoxicillin-clavulanic acid, aligning with studies from Dessie and Addis Ababa, Ethiopia^44,45^. *E. coli* exhibited significant resistance to ampicillin, similar to Nigerian and Ethiopian studies^16,36^, and *K. pneumoniae*, the second most common uropathogen, was fully resistant to ampicillin. While susceptibility was higher to antibiotics like nitrofurantoin and cefpodoxime, both antibiotics were not routinely available in health centers and were difficult to procure in the study catchment area. On a few occasions for the study, a supply was shipped from Addis Ababa to study sites for study participant treatment. This highlights the problem of effectively treating UTIs in primary care settings in Amhara. These findings underscore the urgent need for understanding antimicrobial susceptibility patterns in the specific region and context and improving antibiotic access to medications safe in pregnancy to effectively treat UTIs during pregnancy, such as nitrofurantoin, in resource-limited settings.

In low-resource settings, routine screening of all pregnant women by urine culture is often not feasible given resources, laboratory capacity, and cost. As a result, UTI is typically identified by maternal symptoms or urine dipstick tests in resource-limited settings. Effective UTI screening requires high sensitivity to identify cases needing treatment. We found that dipstick tests for nitrites and leukocyte esterase, both individually and in combination, had very poor sensitivity despite high specificity, consistent with findings from Ethiopia, Kenya, and Ghana^15,46,47^. While combined dipstick results (“NIT + or LE+”) had higher sensitivity, they still did not pick up almost two out of three UTIs. Symptom-based identification of UTIs also showed poor sensitivity, only identifying about half of UTIs. Therefore, our results corroborate the limited value of a symptom or dipstick-based approach to UTI diagnosis, reiterating previous findings^15,48,49^. The low sensitivity in urine dipstick tests results in false negative and missed cases, delaying diagnosis and appropriate treatment, which can lead to severe complications. Moreover, a low PPV for leukocyturia and clinical symptoms reduces diagnostic precision, increasing the likelihood of false positives^15^. This, in turn, leads to overdiagnosis and overtreatment, contributing to antimicrobial resistance and adverse medication effects^50^. Therefore, the difficulty of conducting urine cultures in similar settings highlights the urgent need for more accessible and accurate rapid and low-cost diagnostic tools to improve UTI management and maternal health outcomes.

To our knowledge, this study is one of the first population-based studies on UTI in pregnant women from rural health centers in the Amhara region. The use of the BACTEC-2 system for pathogen identification and antimicrobial susceptibility testing is a notable strength, offering better sensitivity and minimizing technical errors than traditional methods such as morphological identification and disk diffusion AST. However, this study had some limitations. The cross-sectional study design and sampling < 24 weeks of gestation may have underestimated the overall UTI burden, and consequently, the low prevalence may have limited the study’s statistical power to detect significant risk factors during pregnancy. Lastly, the lack of availability of certain AST testing, such as azithromycin and ceftriaxone, restricted the assessment of AST patterns for all potential treatment options in the primary care settings of the study areas.

## Conclusions

In this study, the overall prevalence of UTIs among pregnant women in rural Amhara, Ethiopia was 3.5%. *E. coli* was the predominant uropathogen followed by *K. pneumoniae* and *E. faecalis.* A high level of resistance was observed against commonly used first-line antibiotics such as amoxicillin-clavulanic acid and ampicillin. Maternal age showed borderline association with UTI risk, and urine dipstick tests had limited diagnostic accuracy for detecting UTIs in pregnant women. Therefore, this study underscores the need for improved, affordable UTI diagnostic tools and better access to effective antibiotics to manage UTIs during pregnancy in resource-limited settings.

## Supplementary Information

Below is the link to the electronic supplementary material.

Supplementary Material 1

## Data Availability

In accordance with NIH policy and IRB approvals and data sharing agreements for the ENAT parent study, the quality-controlled de-identified data that support the findings of this study will be made available for up to 5 years from the time of publication, based on a reasonable request from researchers. These data are located in controlled-access data storage at Brown University.
