# Supplementary material for: Prevalence, bacterial etiology, and antimicrobial susceptibility patterns of urinary tract infections among pregnant women in rural West Amhara, Ethiopia
Source: Sci Rep. 2025 Nov 7;15:39040. doi: 10.1038/s41598-025-25655-4 (PMC12594989; doi:10.1038/s41598-025-25655-4)
Supplement: Supplementary file 1 — Supplementary Material 1 [file 41598_2025_25655_MOESM1_ESM.docx]

**Prevalence, bacterial etiology, and antimicrobial susceptibility patterns of urinary tract infections among pregnant women in rural West Amhara, Ethiopia**

Mulatu Melese Derebe, MSc^1,2†^, Unmesha Roy Paladhi, PhD, MPH^3^, Firehiwot Workneh, PhD^4^, Abaineh Munshea, PhD^1^, Gizachew Yismaw, PhD^2^, Kalkidan Yibeltal, MD, MPH^5^, Nebiyou Fasil, PhD, MPH^6^, Alemayehu Worku, PhD^4^, Tsehaynesh Gebreyesus, MSc^2^, Wudu Tafere, MSc^2^, Alem Tsega, MSc^2^, Parul Christian, PhD^7^, Rose L. Molina, MD, MPH,^8^ Blair J. Wylie, MD^8^, Yemane Berhane, MD, MPH, PhD^4^*, Anne CC Lee, MD, MPH^3^*

^1^Health Biotechnology Division, Institute of Biotechnology, Bahir Dar University, Bahir Dar, Ethiopia

^2^Amhara Public Health Institute, Bahir Dar, Ethiopia

^3^Global Alliance for Infant and Maternal Health, Warren Alpert Medical School, Department of Pediatrics, Brown University, Providence, RI, USA

^4^Department of Epidemiology and Biostatistics, Addis Continental Institute of Public Health, Addis Ababa, Ethiopia

^5^Department of Reproductive Health and Population, Addis Continental Institute of Public Health, Addis Ababa, Ethiopia

^6^Department of Global Health and Health Policy, Addis Continental Institute of Public Health, Addis Ababa, Ethiopia

^7^Department of Human Development, Teachers College, Columbia University, New York, USA

^8^Beth Israel Deaconess Medical Center, Boston, MA

*Co-senior authors

†Corresponding author: Mulatu Melese Derebe, MSc
Institute of Biotechnology, Bahir Dar University, Bahir Dar, Ethiopia
[**mulatumaciph@gmail.com**](mailto:mulatumaciph@gmail.com)

**Supplementary documents:**

**Supplementary Table S1:** The etiology of uropathogens isolated from symptomatic and asymptomatic pregnant women diagnosed with UTIs in rural West Amhara, Ethiopia, (2020–2022).

| Bacterial Isolates | Asymptomatic  N= 12 | Symptomatic  N=9 | Total  N=21 |
| --- | --- | --- | --- |
| *Escherichia coli* | 5 (41.7%) | 7 (58.3%) | 12 (57.1%) |
| *Enterococcus faecalis* | 3 (100.0%) | 0 (0.0) | 3 (1.3%) |
| *Klebsiella pneumoniae* | 2 (66.7%) | 1 (33.3%) | 3 (1.3%) |
| *Staphylococcus aureus* | 0 (0.0) | 1 (100.0%) | 1 (4.8%) |
| *Staphylococcus saprophyticus* | 1 (100.0%) | 0 (100.0%) | 1 (4.8%) |
| *Other streptococcus species* | 1 (100.0%) | 0 (0.0) | 1 (4.8%) |
| % | 12 (57.1%) | 9 (42.9%) | 21 (100.0%) |

**Supplementary Figure S1**. Antimicrobial susceptibility patterns of UTI uropathogens (n=21) from urine culture among pregnant women receiving ANC visits in the rural West Amhara, Ethiopia (2020–2022).

**
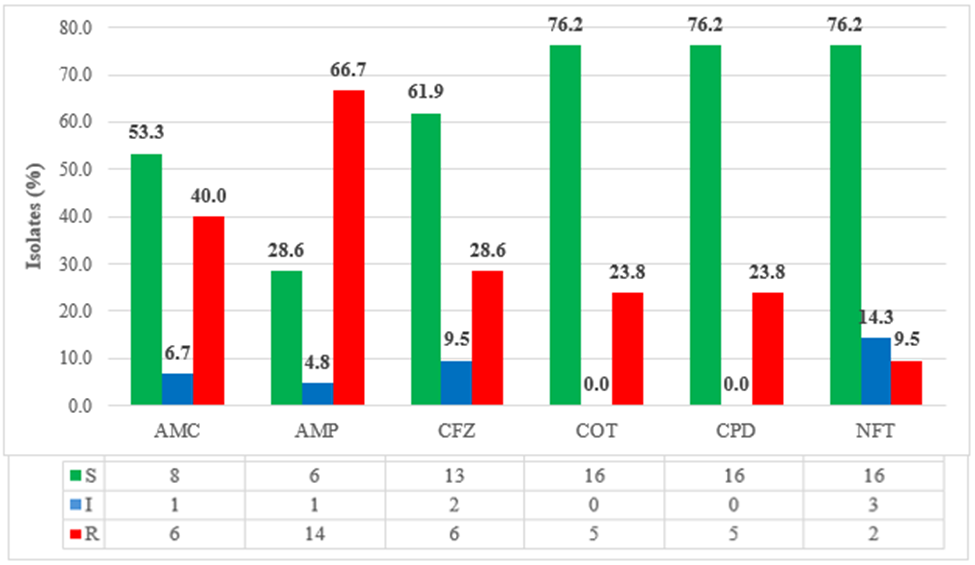
**

Abbreviations: AMC- amoxacillin-clavilanic acid; AMP- ampicillin; CFZ- cefazolin; COT- cotrimoxazole; CPD- cefpodoxime; NFT- nitrofurantoin.
